# Bone cement distribution is a potential predictor to the reconstructive effects of unilateral percutaneous kyphoplasty in OVCFs: a retrospective study

**DOI:** 10.1186/s13018-018-0839-5

**Published:** 2018-06-07

**Authors:** Jiachen Lin, Lie Qian, Changqing Jiang, Xiuyuan Chen, Fan Feng, Lifeng Lao

**Affiliations:** Department of Orthopaedic Surgery, Renji Hospital, School of Medicine, Shanghai Jiao Tong University, 160 Pujian Road, Shanghai, 200127 China

**Keywords:** Osteoporotic vertebral compression fracture, Unilateral percutaneous kyphoplasty, Vertebral height restoration, Cobb angle, Bone cement extravasation

## Abstract

**Background:**

Osteoporotic vertebral compression fracture **(**OVCF) is a common type of fracture, and percutaneous kyphoplasty (PKP) is an eligible solution to it. Previous studies have revealed that both the volume and filling pattern of bone cement correlate with the clinical outcomes after PKP procedure. However, the role of bone cement distribution remains to be illustrated.

**Methods:**

To retrospectively evaluate the relationship between the bone cement distribution and the clinical outcomes of unilateral PKP, we enrolled 73 OVCF patients receiving unilateral PKP treatment. All the intervened vertebrae were classified into three groups based on the bone cement distribution observed on postoperative X-ray films. Preoperative and postoperative radiographic parameters including the vertebral height and kyphotic Cobb angle were recorded, and anterior vertebral height restoration rate (AVHRR) and Cobb angle correction (CR) were then calculated to assess the vertebral height reconstruction. Preoperative and postoperative Oswestry Disability Index (ODI) and visual analogue scale (VAS) were adopted by interviewing patients to assess the mobility improvement and pain relief. Demographic data, body mass index (BMI), lumbar bone mineral density (evaluated by BMD T-score) of each patient, bone cement volume (BV), and bone cement extravasation (BE) were also recorded. Between- and within-group comparisons and multivariable correlation analysis were carried out to analyze the data.

**Results:**

VAS and ODI scores were both significantly improved in all of the enrolled cases with no significant differences between groups. Among the three groups, the average age, AVHRR, and BV were significantly different. Occurrence of BE was significantly different between two of the three groups. AVHRR was demonstrated to correlate negatively with preoperative anterior vertebral height ratio and positively with preoperative Cobb angle, CR, diffusion score, and ODI changes.

**Conclusions:**

Bone cement distribution is a potential predictor to the reconstructive effects in unilateral PKP for OVCFs. Bone cement distribution is associated with AVHRR and BV, as well as the risk of BE occurrence. Greater bone cement distribution may indicate better vertebral restoration along with a higher BE risk.

## Background

Osteoporotic vertebral compression fracture (OVCF) is a common type of fracture. The prevalence of OVCF was reported as high as 20% in people aged over 50 [1]. Along with the aging of population, the morbidity and disability caused by OVCFs has been continuously burdening the global society [2–4]. The clinical features of OVCF mainly include acute or chronic back pain and physical disability, as well as the distinctive radiographic finding of “wedge-shaped” vertebral bodies due to mechanical compression. The clinical management of OVCFs aims at restoration of both the physical structure and biomechanical resistance of the impaired spinal segment.

As an eligible solution to OVCFs, the percutaneous kyphoplasty (PKP) has demonstrated great efficiency in pain relief and functional improvement [5–8]. Notably, it is common that the intravertebral bone cement distribution after unilateral PKP procedure varies among different vertebral bodies during the clinical practice. Besides, previous studies revealed that the bone cement volume (BV) and filling patterns correlate with surgical complications and curative effects [9–13]. However, the therapeutic implication of bone cement distribution in unilateral PKP remains to be illustrated.

In this study, we classified the fractured vertebrae in OVCF patients into three groups according to the bone cement distribution recorded on standing anteroposterior radiographs after unilateral PKP implementation. We compared the radiographic findings and clinical outcomes throughout the three groups and further evaluated the relationship between the bone cement distribution and those parameters.

## Methods

### Patients

A total number of 82 vertebrae in 73 patients were included as subjects. All patients were diagnosed with OVCFs and accepted unilateral percutaneous kyphoplasty [14, 15] in our department from January 2012 to January 2016 and were followed up for at least 1 year after surgery. General health evaluation was performed to rule out surgical contraindication before the procedure. All patients had been suffering from intolerable pain (VAS ≥ 6) despite conservative therapy for 6 to 12 weeks. Exclusive criteria include the following: extra internal fixation implementation, vertebral fracture caused by metastatic cancer, mental instability, major cardiopulmonary insufficiency, and loss to follow-up. The study was approved by the ethic committee of Renji Hospital. All enrolled patients gave informed consent.

Demographic data including age, gender, height, weight, bone mineral density (BMD, T-score), time to surgery, and levels of injury were recorded. Preoperative, postoperative, and following up standing X-ray plain films were taken, and preoperative dual-energy X-ray absorptiometry was performed and recorded. Patients were interviewed and evaluated by physicians who did not participate in the following surgical procedure. Visual analogue score (VAS) and Oswestry Disability Index (ODI) were adopted to interpret pain intensity and disability before the surgery.

### Surgical procedure

All patients received unilateral PKP in prone position under the guidance of C-arm fluoroscopy after local anesthesia with 1% lidocaine. An 11- to 15-gauge biopsy needle was inserted through the unilateral vertebral pedicle. A balloon was then inserted along the working cannula and inflated with radiocontrast agent until the intraluminal pressure reached around 12 atm. After balloon was retrieved, formulated polymethyl methacrylate (PMMA) mixture was instilled into the intravertebral cavity as bone cement material. According to vender recommendation, the filling PMMA was mixed in a powder-to-liquid ratio of 20:9.4. The volume of PMMA usage (BV) was recorded by the operating surgeons. All enrolled patients underwent uneventful procedure and were encouraged to ambulate 3 h after surgery.

### Radiographic findings

Standing anteroposterior and lateral X-ray films of thoracolumbar spine region were taken before and 24 h after the surgery. The morphological parameters of fractured vertebrae, together with bone cement distribution and extravasation were observed and recorded.

**Fig. 1:**
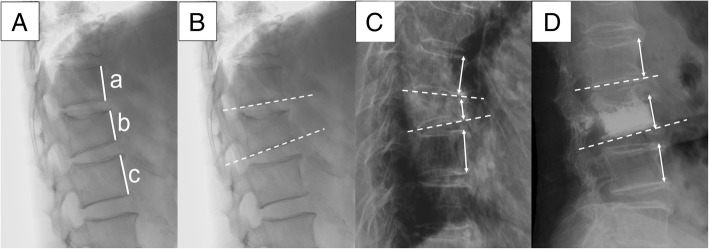
**A**–**D** Radiographic evaluation of compressed vertebrae. **A** Anterior vertebral height ratio (AVHR) was calculated as percentile of anterior vertebral height of the compressed vertebra (b), divided by the mean anterior vertebral height of the adjacent upper and lower vertebrae (a + c)/2. The anterior vertebral height restoration rate (AVHRR) = preoperative AVHR − postoperative AVHR. **B** Cobb angle was determined as the degree between the lines of lower endplate of compressed vertebra and the adjacent upper vertebra. The Cobb angle correction (CR) = preoperative Cobb angle − postoperative Cobb angle. **C**–**D** Examples of preoperative (**C**) and postoperative (**D**) images for evaluating vertebral restoration. The X-ray films were taken 1 day before and after the surgery, respectively, from an 80-year-old female patient with L3 compression fracture. Both the AVHRR (35.5% = 87.3–51.8%) and CR (11.3° = 16.1°–4.8°) are significant after treated by unilateral PKP

The deformity of vertebrae was evaluated by anterior vertebral height ratio (AVHR) and kyphotic Cobb angle. The anterior vertebral height ratio was calculated as percentile of anterior vertebral height of the compressed vertebra against the mean anterior vertebral height of adjacent upper and lower vertebra [10] (Fig. 1A). The kyphotic Cobb angle was defined as the degree between the lower endplate of compressed vertebra and adjacent upper vertebra (Fig. 1B). The anterior vertebral height restoration rate (AVHRR) was measured as the difference between the preoperative and postoperative AVHRs while the correction of kyphosis was measured as the difference between preoperative and postoperative Cobb angles. An example of evaluating vertebral reconstruction by measuring pre- and postoperative radiographic parameters was presented in Fig. 1C–D.

**Fig. 2:**
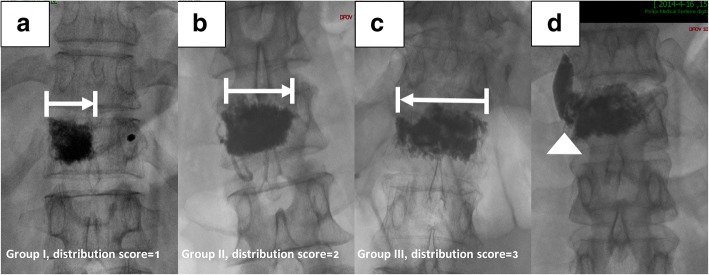
**a**–**d** Observation of bone cement distribution and classification of vertebrae. **a** The vertebrae in which the bone cement located unilaterally (restricted by the midline) comprised group I. **b** The vertebrae in which the bone cement diffused across the midline but not to the contralateral vertebral pedicle comprised group II. **c** The vertebrae in which bone cement diffused over the contralateral pedicle comprised group III. **d** Existence of bone cement extravasation was recorded

The included vertebrae were then divided into three groups according to the bone cement distribution. The vertebrae in which the bone cement distribution was restricted unilaterally comprised the group I (the bone cement did not pass the midline of the vertebra, Fig. 2a). The vertebrae in which the bone cement distributed across the midline but not to the contralateral vertebral pedicle comprised the group II (Fig. 2b). The vertebrae in which bone cement distributed over the contralateral pedicle comprised the group III (Fig. 2c). We then quantified bone cement distribution by using diffusion score (DS). The DS in group I is 1, and the DS in groups II and III are 2 and 3, respectively. Existence of bone cement extravasation (BE) was also recorded (Fig. 2d).

### Clinical outcomes

Being blinded to the subjective categorization, surgeons assessed the patients at 24 h, 3 days, 1 month, 3 months, and 1 year after surgery. All patients completed the VAS and ODI questionnaire before the surgery, 1 day after the surgery, and at following up to evaluate pain reliefs and functional recovery conditions. No recurrence of symptomatic adjacent vertebral fracture was revealed in this retrospective case series.

### Statistical analysis

Software IBM SPSS Statistics 23.0 was adopted on data analysis. Level of statistical significance was defined as *p* value < .050. Analysis of variance (ANOVA) with Bonferroni and Tamhane correction was used to compare the difference of age, BMI, BMD, preoperative AVHR and Cobb angle, and surgical CR among groups. The Kruskal-Wallis one-way ANOVA test was performed to distinguish the difference of BV and AVHRR among groups. The chi-square test was conveyed to analyze the difference of occurrence of BE. The Wilcoxon test was performed for within-group comparisons of VAS and ODI scores. Multivariable correlation analysis of age, gender, BMI, BMD, preoperative VHR and Cobb angle, AVHRR, CR, BV, and BE occurrence was analyzed by Spearman correlation coefficient test.

## Results

### Demographic data

Of 73 patients, 15 were male and 58 were female, aging 70.99 ± 8.53 (56–95, Table 1). A total number of 82 subjective vertebrae were subdivided into three groups according to bone cement distribution condition as mentioned. Age, gender, BMI, BMD T-score, thoracic, and lumber spinal vertebrae ratio were demonstrated in Table 1. There was a significant difference of average age among the three groups (*p* = .040). The average age of group III (73.23 ± 8.56 years) was significantly higher than that of the group II (68.18 ± 9.10 years, *p* = .023), but not significantly higher than that of the group I (68.94 ± 6.21 years, *p* = .075). The average age difference was of no statistical significance between group I and group II (*p* = .778).

**Table 1:** Demographic data

Parameters	Group I	Group II	Group III	*P* value
*N*	17	22	43	NA
Age (years)	68.94 ± 6.21	68.18 ± 9.10	73.23 ± 8.56	.040*
Gender (M:F)	6/17	4/22	6/43	NA
BMI (kg/m^2^)	23.32 ± 1.51	22.88 ± 1.96	22.85 ± 2.07	.684
BMD (T-score)	− 3.22 ± 0.52	− 3.17 ± 0.68	− 3.31 ± 0.54	.627
T spine ratio	4/17	7/22	15/43	NA
L spine ratio	13/17	15/22	28/43	NA

The average age of group III is significantly higher than that of the group II (*p* = .023) but not significantly higher than that of the group I (*p* = .075)

*F* female, *M* male, *BMI* body mass index, *BMD* bone mineral density, *NA* not applicable

*P value by analysis of variances

### Radiographic parameters

**Fig. 3:**
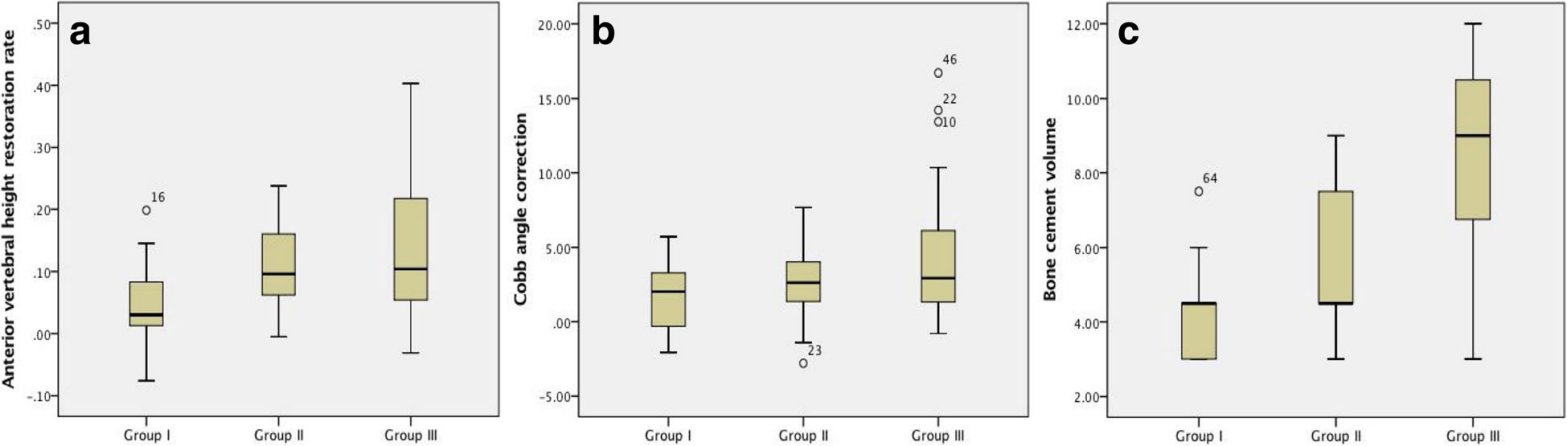
**a**–**c** Comparisons of the anterior vertebral height restoration rate (**a**), Cobb angle correction (**b**), and bone cement volume (**c**) among the three groups

As for the preoperative parameters, no statistically significant difference of preoperative AVHR and kyphotic Cobb angle was found. AVHRR was revealed to differ among all groups (.043 ± .066, .106 ± .069, and .130 ± .110, respectively, *p* = .007, Kruskal-Wallis test, Table 2, Fig. 3a). Notably, the AVHRR of group I was significantly lower than the other two groups (*p* = .007 and .000, respectively), while the difference was nonsignificant between group II and group III (*p* = .280). CR differed among groups but with no statistical significance (1.66 ± 2.47, 2.61 ± 2.42, 4.24 ± 4.08, respectively, *p* = .089, Kruskal-Wallis test, Table 2, Fig. 3b). However, CR in group III was significantly higher than in group I (*p* = .019) and group II (*p* = .048).

**Table 2:** Radiographic parameters

Parameters	Group I	Group II	Group III	*P* value
Pre-op AVRR	.792 ± .176	.753 ± .171	.696 ± .148	.092
Post-op AVHR	.835 ± .159	.859 ± .175	.826 ± .103	.546
Anterior VHRR	.043 ± .066	.106 ± .069	.130 ± .110	.007*
Group I–II				.007*
Group I–III				.000*
Group II–III				.280
Pre-op CA (°)	6.51 ± 11.47	6.84 ± 8.05	10.30 ± 8.37	.200
Post-op CA (°)	4.84 ± 11.33	4.23 ± 7.82	6.07 ± 6.82	.672
CR	1.66 ± 2.47	2.61 ± 2.42	4.24 ± 4.08	.089
Group I–II				.241
Group I–III				.019*
Group II–III				.048*
BV (ml)	4.15 ± 1.25	5.46 ± 2.05	8.43 ± 2.20	.000*
Group I–II				.000*
Group I–III				.000*
Group II–III				.000*
BE occurrence	0/17	4/22	16/43	NA
Group I–II				.140
Group I–III				.016*
Group II–III				.240

*Pre-op* preoperative, *Post-op* postoperative, *AVHR* anterior vertebral height ratio, *CA* kyphotic Cobb angle, *AVHRR* anterior vertebral height restoration rate, *CR* Cobb angle correction, *BV* bone cement volume, *BE* bone cement extravasation, *NA* not applicable

*P value by analysis of variances and multiple comparisons, independent sample *T* test, Kruskal-Wallis one-way ANOVA test, and chi-square test

BV differed significantly among all groups (4.15 ± 1.25 ml, 5.46 ± 2.05 ml, and 8.43 ± 2.20 ml respectively, *p* = .000, Table 2, Fig. 3c). BV of group III was higher than that of the other two groups, and that of group II was higher than that of group I, all with significance.

BE of group III (16/43 = 0.372, Table 2) was significantly higher than that of the group I (0/17 = .000, *p* = .016). BE occurrence in group II (4/22 = .182) was greater than that in group I, and that in group III was greater than that in group II, but the difference was nonsignificant (*p* = .140 and .240, respectively).

### Correlations between parameters

The body mass index (BMI) showed no difference among the three groups (23.32 ± 1.51, 22.88 ± 1.96, and 22.85 ± 2.07, respectively, *p* = .684, Table 1). No correlation was found between BMI and BV, preoperative AVHR, AVHRR, preoperative CA, CR, diffusion score, changes of VAS, and ODI after surgery (Spearman correlation coefficient, Table 3).

**Table 3:** Correlations of parameters with anterior vertebral height restoration rate

Factors	Correlation coefficient	*P* value
Age	.141	.205
Gender	− .065	.562
BMI	− .039	.729
BMD (T-score)	− .164	.140
Pre-op AVHR	− .518	.000*
Pre-op CA	.327	.003*
CR	.716	.000*
BCV	.172	.123
Diffusion score	.300	.006*
VAS changes	.046	.669
ODI changes	.250	.024*

AVHRR correlates positively with preoperative CA, CR, diffusion score, and ODI changes, while negatively with preoperative AVHR with statistical significance

*BMI* body mass index, *BMD* bone mineral density, *Pre-op* preoperative, *Post-op* postoperative, *AVHR* anterior vertebral height ratio, *CA* kyphotic Cobb angle, *AVHRR* anterior vertebral height restoration rate, *CR* Cobb angle correction, *BV* bone cement volume, *BE* bone cement extravasation, *VAS* visual analogue score, *ODI* Owsertry Disability Index

*P value by Spearman correlation coefficient

The bone mineral density (BMD T-score) showed no difference among the three groups (− 3.22 ± 0.52, − 3.17 ± 0.68, and − 3.31 ± 0.54 respectively, *p* = .627, Table 1). No correlation was found between BMD and BV, AVHR, AVHRR, preoperative CA, CR, diffusion score, changes of VAS, and ODI after surgery (Spearman correlation coefficient, Table 3).

AVHRR correlates with preoperative AVHR negatively (Spearman correlation coefficient, *r* = − .518, *p* < .001, Table 3). AVHRR also showed significant positive correlations with preoperative CA, CR, diffusion score, and ODI changes after surgery (Spearman correlation coefficient, Table 3). No correlation was found between AVHRR and VAS changes or BV.

### Clinical outcomes

**Fig. 4:**
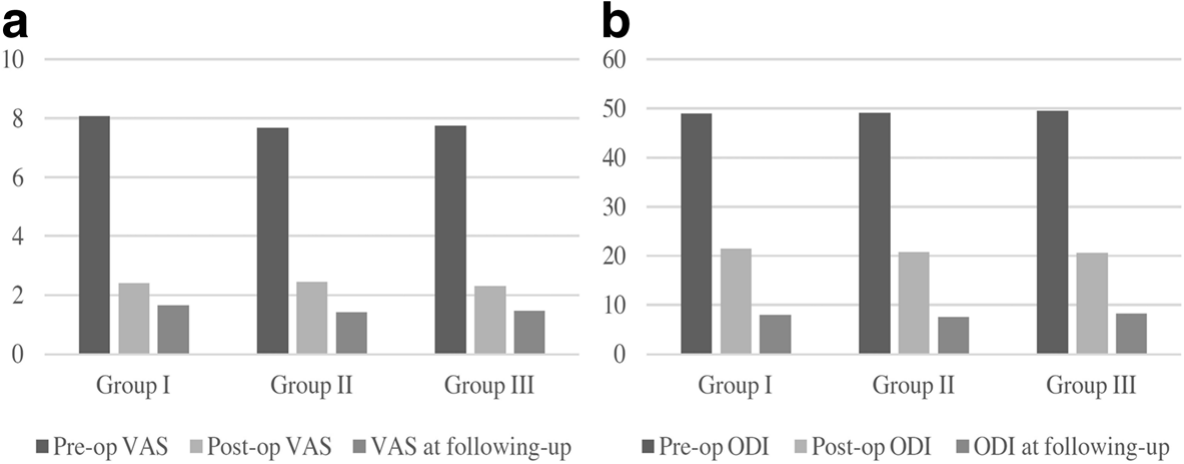
**a**–**b** Clinical outcomes data both VAS (**a**) and ODI (**b**) scores were significantly improved 1 day after the surgery and during the follow up. No significant difference among the three groups was revealed

The VAS and ODI were both significantly improved 24 h after the surgery and during the following up (*p* < .001, Table 4, Fig. 4a–b). However, there was no significant difference of pre- and post-operative and following-up clinical outcome measures among the three groups.

**Table 4:** VAS and ODI changes

Parameters	Group I	Group II	Group III
Pre-op VAS	8.06 ± 1.03	7.68 ± .839	7.77 ± .751
Post-op VAS	2.41 ± .507	2.45 ± .858	2.30 ± .708
VAS at following-up	1.65 ± .702	1.41 ± .503	1.47 ± .550
Pre-op/post-op VAS (*P* value)	.000*	.000*	.000*
Post-op VAS/following up VAS (*P* value)	.001*	.000*	.000*
Pre-op ODI	48.94 ± 3.15	49.14 ± 3.76	49.58 ± 3.40
Post-op ODI	21.47 ± 1.94	20.82 ± 3.89	20.63 ± 3.11
ODI at following up	7.88 ± 1.62	7.45 ± 1.26	8.26 ± 1.94
Pre-op/post-op ODI (*P* value)	.000*	.000*	.000*
Post-op ODI/following up ODI (*P* value)	.000*	.000*	.000*

VAS and ODI scores improved significantly after surgery and at following up. However, there was no significant difference of the changes among the three groups

*Pre-op* preoperative, *Post-op* postoperative, *VAS* visual analogue score, *ODI* Oswestry Disability Index

*P value by Wilcoxon matched-pair signed-rank test

## Discussion

Cement augmentation vertebroplasty was initially introduced into vertebral fracture treatment by French physicians in 1987 [16]. The technique was improved by using balloon inflation to create larger intervertebral space in order to achieve better restoration and prevent bone cement extravasation, which was known as percutaneous kyphoplasty [17]. Radiographic evaluation plays an important role in assessment of PKP treatment. Intraoperative fluoroscopy avails surgeons to observe the position of invading needle, as well as the process of instillation and distribution of the bone cement. Therefore, further understanding of the relationship between intraoperative radiographic findings and the therapeutic outcomes may help optimize surgical decision. Several studies have uncovered the effects of bone cement volume on PKP efficacy [11, 12, 18], but few emphasized on bone cement distribution or filling pattern [13]. In this study, the correlation between bone cement distribution and vertebral reconstruction in unilateral PKP was revealed, shedding light on further improvement of the procedure.

PKP implementation could achieve great curative effect in OVCFs with instant pain relief and excellent functional recovery, as well as significant vertebral height restoration and kyphotic deformity correction [5, 6, 19–21]. Consistent with former studies, the vertebral height restoration and kyphotic Cobb angle correction were significant after the surgery in this study. The VAS and ODI scores were also greatly improved, while between-group comparison showed no significant difference.

Anterior vertebral height restoration rate was significantly different among groups, and it increased along with the bone cement expansion (group III > group II > group I, *p* = .007, Table 2). The change of Cobb angle was consistent with the change of anterior and central vertebral height ratio. Notably, AVHRR was relatively minimal in group I compared to group II and III. We hypothesized this phenomenon was of predictive significance. Besides, the difference of AVRHH between group II and III was nonsignificant. Therefore, we suggest that when bone cement distribution is wide enough (i.e., spread over the area between middle line and contralateral vertebral pedicle), the unilateral PKP is able to provide the more significant vertebral height restoration. Previously, there was a dispute on the comparison between unilateral and bilateral kyphoplasty [22–25]. Since the unilateral PKP procedure can produce similar bone cement distribution in group III as bilateral PKP procedure, we assume that it is the bone cement distribution that decides the therapeutic effect of cementoplasty.

The correlation study indicated that AVHRR was positively correlated with preoperative compression rate (i.e., preoperative vertebral height ratios, Cobb angle), which was supported by Lee et al. [26], who had discovered that anterior vertebral height was higher in 70% collapsed vertebrae compared to collapsed 50~ 70% group and collapsed 30~ 50% group. Data also suggested that the structural disruption of cancellous bone and the endplate fracture may provide larger potential space for cement instillation in severe fracture comparing to mild ones, leading to higher AVHRR. To be noted, previous studies [13, 26] also suggest that uneven distribution of bone cement may undermine the physical strength of “non-PMMA supported area,” leading to recurrent fractures.

Bone cement volume was revealed to increase as bone cement distribution expanded (Fig. 3c). No cement extravasation was found in group I. However, occurrence of extravasation raised in group II and group III as bone cement built up, and the difference between group I and III was significant (*p* = .016, Table 2). It has been suggested that higher BV is one of the risk factors for bone cement extravasation [11]. Li et al. [10] also indicated that the upper endplate fracture may induce bone cement leakage in PKP. We hypothesized that in group I, restricted spread of bone cement distribution, as well as lower BV, together contributed to prevent BE occurrence. Therefore, bone cement distribution may be a predictive risk factor to BE.

This retrospective study had several limitations. First of all, considering the fact that some of the OVCF patients admitted to our department were allocated to bilateral PKP treatment, the sample size was not large as expected. Secondly, other factors such as endplate rupture [10] may also compromise the therapeutic outcomes. Last but not least, the CT scanning and reconstruction was supposed to be more illustrative when evaluating vertebral deformity; however, most of the enrolled patients did not accept postoperative CT. Further prospective and optimized study is required in the future.

## Conclusion

Bone cement distribution is associated with the reconstructive effect in the unilateral percutaneous kyphoplasty. Greater bone cement distribution may indicate greater anterior vertebral height restoration and Cobb angle correction, which is also demanding more bone cement usage and extra monitor on bone cement extravasation. As an intraoperative radiographic finding, the bone cement distribution could become a predictive factor in evaluating the therapeutic reconstruction efficacy of unilateral PKP.

## Abbreviations

AVHR: Anterior vertebral height restoration
AVHRR: Anterior vertebral height restoration rate
BE: Bone cement extravasation
BMD: Bone mineral density
BMI: Body mass index
BV: Bone cement volume
CA: Cobb angle
CR: Cobb angle correction
NA: Not applicable
ODI: Oswestry disability index
OVCF: Osteoporotic vertebral compression fracture
PKP: Percutaneous kyphoplasty
PMMA: Polymethyl methacrylate
Post-op: Postoperative
Pre-op: Preoperative
VAS: Visual analogue score
